# The Fluid Aspect of the Mediterranean Diet in the Prevention and Management of Cardiovascular Disease and Diabetes: The Role of Polyphenol Content in Moderate Consumption of Wine and Olive Oil

**DOI:** 10.3390/nu11112833

**Published:** 2019-11-19

**Authors:** Paola Ditano-Vázquez, José David Torres-Peña, Francisco Galeano-Valle, Ana Isabel Pérez-Caballero, Pablo Demelo-Rodríguez, José Lopez-Miranda, Niki Katsiki, Javier Delgado-Lista, Luis A. Alvarez-Sala-Walther

**Affiliations:** 1 Internal Medicine, Hospital General Universitario Gregorio Marañón, 28007 Madrid, Spain; ditanopaola@gmail.com (P.D.-V.); paco.galeano.valle@gmail.com (F.G.-V.); pbdemelo@hotmail.com (P.D.-R.); 2 Lipids and Atherosclerosis Unit, Department of Internal Medicine, Maimonides Biomedical Research Institute of Cordoba (IMIBIC), Reina Sofia University Hospital, University of Cordoba, Av. Menéndez Pidal s/n, 14004 Cordoba, Spain; azarel_00@hotmail.com (J.D.T.-P.); md1delij@uco.es (A.I.P.-C.); md1lomij@uco.es (J.L.-M.); 3 CIBER Fisiopatología de la Obesidad y Nutrición (CIBEROBN), Instituto de Salud Carlos III (ISCIII), 28007 Madrid, Spain; 4 Instituto de investigación sanitaria Gregorio Marañón, 28007 Madrid, Spain; 5 Department of Medicine, School of Medicine, Universidad Complutense de Madrid, 28007 Madrid, Spain; 6 First Department of Internal Medicine, Division of Endocrinology and Metabolism, Diabetes Center, Medical School, AHEPA University Hospital, Thessaloniki 54621, Greece; nikikatsiki@hotmail.com

**Keywords:** mediterranean diet, cardiovascular disease, diabetes, polyphenols, wine, olive oil

## Abstract

A growing interest has emerged in the beneficial effects of plant-based diets for the prevention of cardiovascular disease, diabetes and obesity. The Mediterranean diet, one of the most widely evaluated dietary patterns in scientific literature, includes in its nutrients two fluid foods: olive oil, as the main source of fats, and a low-to-moderate consumption of wine, mainly red, particularly during meals. Current mechanisms underlying the beneficial effects of the Mediterranean diet include a reduction in inflammatory and oxidative stress markers, improvement in lipid profile, insulin sensitivity and endothelial function, as well as antithrombotic properties. Most of these effects are attributable to bioactive ingredients including polyphenols, mono- and poly-unsaturated fatty acids. Polyphenols are a heterogeneous group of phytochemicals containing phenol rings. The principal classes of red wine polyphenols include flavonols (quercetin and myricetin), flavanols (catechin and epicatechin), anthocyanin and stilbenes (resveratrol). Olive oil has at least 30 phenolic compounds. Among them, the main are simple phenols (tyrosol and hydroxytyrosol), secoroids and lignans. The present narrative review focuses on phenols, part of red wine and virgin olive oil, discussing the evidence of their effects on lipids, blood pressure, atheromatous plaque and glucose metabolism.

## 1. Introduction

A growing interest has emerged on the beneficial effects of plant-based diets for the prevention of chronic diseases, including cardiovascular disease (CVD), type 2 diabetes mellitus (T2DM) and obesity [1]. The Mediterranean diet (MedDiet), described by Keys in the 1960s, is one of the most widely described and evaluated dietary patterns in scientific literature. The Seven Countries study, including the USA, Japan, Northern and Southern European cohorts, has been the cornerstone of health-promoting nutrition, highlighting the importance of the MedDiet [2]. The MedDiet refers to the dietary pattern of people living in the Mediterranean Sea. Traditionally, it is characterized by high intakes of vegetables, legumes, fruits, nuts, grains, fish, seafood and poultry as sources of protein, olive oil, nuts and low-to-moderate intake of red wine, as well as low intake of dairy products, red and processed meat, cream, and sugar drinks [3,4]. The MedDiet consists of a low saturated fat and a high monounsaturated fat content (derived mainly from oleic acid in the olive oil). Lipid sources in the MedDiet mainly include foods rich in unsaturated fatty acids and antioxidants (e.g., olive oil, fish, and nuts) [4,5]. The MedDiet contains two fluid foods: olive oil, as the main source of fats, and a low-to-moderate consumption of wine, mainly red, particularly during meals [4].

A large body of evidence demonstrated an inverse association between adherence to the MedDiet and a lower risk of all-cause mortality, CVD, T2DM, site-specific cancers, and cognitive disorders [6]. The PREDIMED (PREvención con DIeta MEDiterránea) study was a long-term prospective, double blind, controlled, multicenter trial comparing MedDiet supplemented with extra-virgin olive oil or nuts versus a control diet with reduced fat intake in a primary prevention high cardiovascular risk Spanish population [7]. This trial confirmed the beneficial role of MedDiet, suggested in previous epidemiological and prospective cohort studies, since patients in both MedDiet groups (i.e., with extra-virgin olive oil or nuts) had a significantly lower prevalence of CVD events compared with controls [7].

Regular, moderate alcohol consumption, mainly red wine, has been suggested to play a role in CVD prevention. In this context, the French population has a relatively low incidence of coronary heart disease (CHD), while having a diet relatively rich in saturated fats [8]. This so-called ‘French Paradox’ has been related to the moderate but significant intake of wine in this population (267 to 383 mL/day, meaning 23–34 g of wine alcohol) [8]. A recent study pointed out that alcohol consumption is one of the leading factors for global disease burden [9]. Furthermore, based on some studies with inherent limitations, it has been proposed that alcohol, even when consumed moderately, can increase the risk of several diseases [10]. Thus, any suggestion of the possible benefits of wine intake should clearly be circumscribed to a low-to-moderate consumption (i.e., 1–2 drinks/day or ~150–300 mL/day) [11], within a Mediterranean pattern, which means mainly red wine consumed with principal meals [12]. This type of MedDiet wine consumption pattern is considered positive within the PREDIMED Mediterranean Diet Score [13].

Current mechanisms underlying the beneficial effects of the MedDiet include reduction of inflammatory and oxidative stress markers, and an improvement in lipid profile, insulin sensitivity and endothelial function, as well as antithrombotic properties [14]. Most likely, these effects are attributable to bioactive ingredients such as polyphenols, mono- and poly-unsaturated fatty acids (mainly oleic acid from olive oil) or fiber [3].

The aim of the present narrative review is to summarize the evidence regarding the beneficial effects of polyphenols contained in the fluid components of the MedDiet, mainly olive oil and red wine, in terms of CVD risk factors [lipids, blood pressure (BP), endothelial dysfunction] and glucose metabolism.

## 2. Polyphenols and Phenolic Compounds

Polyphenols are common constituents of foods of plant origin and major antioxidants in our diet [15]. Polyphenols are especially abundant in fruits, vegetables, olives, whole grains, legumes, as well as in tea, coffee, olive oil and red wine [16]. In terms of structure, polyphenols have one or more phenolic groups, capable of reducing reactive oxygen species and various organic substrates and minerals. These properties explain the considerable interest in their role in the prevention of several major chronic diseases associated with oxidative stress, including atherogenesis. Physiologically, these molecules are produced to manage environmental stressors affecting plant integrity, such as ultraviolet lights, free radicals, and uncommon temperatures, therefore limiting the effects of oxidative stress [17]. Concerning vegetable products in the Mediterranean basin, olives and grapes are very sensitive to stressors; researchers have demonstrated that stress enhances polyphenol production in both olives and grapes [18].

A mean dietary intake of polyphenols about 1 g per day has been reported in the Finnish population [19], but it is probably much higher in other countries. Polyphenol consumption is much higher than other known dietary antioxidants, such as vitamin C, vitamin E and carotenoids [15].

Polyphenols have been suggested to exert a plethora of biological activities including hormonal regulation and antioxidant, anti-inflammatory, anti-microbial, anti-proliferative and pro-apoptotic effects. There is also increasing evidence that long-term intake has a favorable effect on the incidence of CVD [20] and can reduce the incidence of several cancers and other chronic diseases, including T2DM and neurodegenerative disorders [21].

**Figure 1:**
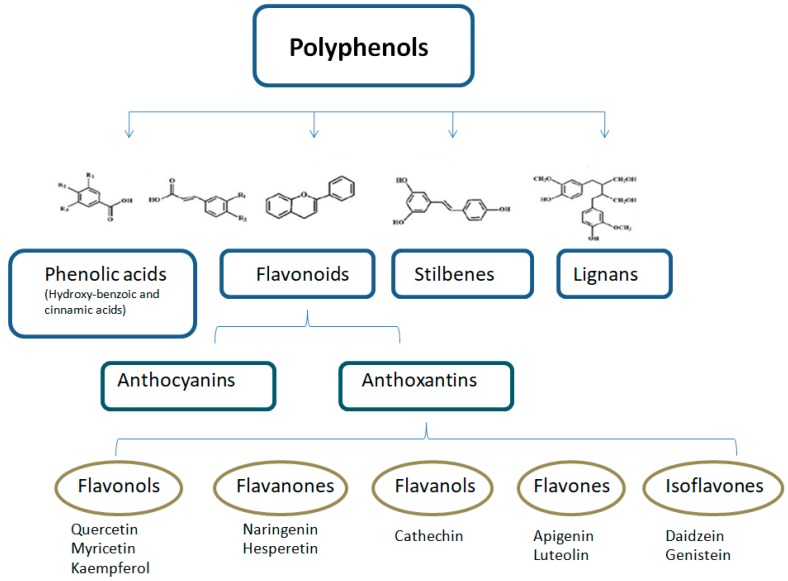
Classification and structure of the main polyphenol classes. Adapted from Pandey KB et al. Oxid. Med. Cell. Longev. 2009, 2, 270–8.

**Figure 2:**
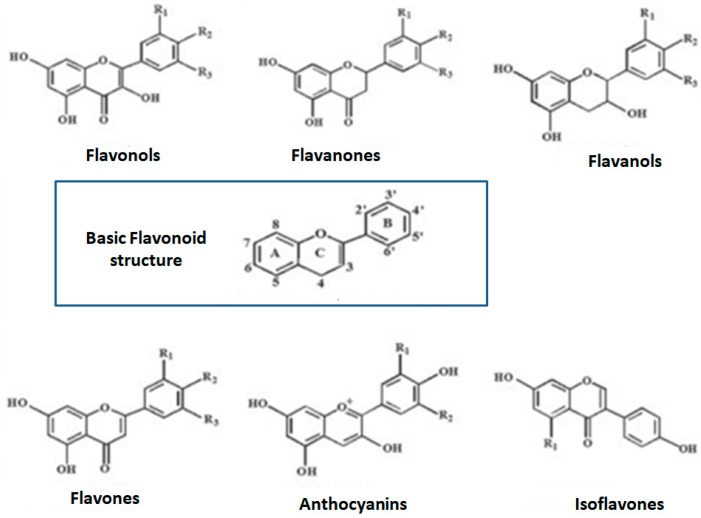
Chemical structures of sub-classes of flavonoids. Adapted from Guasch-Ferré M 2017 [1].

Polyphenols are a large and heterogeneous group of phytochemicals containing phenol rings, which are divided into flavonoids, phenolic acids, stilbenes, and lignans (Figure 1; Figure 2) [1,22]. More than 8000 different polyphenols have been described so far, each one showing differences in their properties and bioavailability [23,24].

## 3. Polyphenols Content in Wine

**Figure 3:**
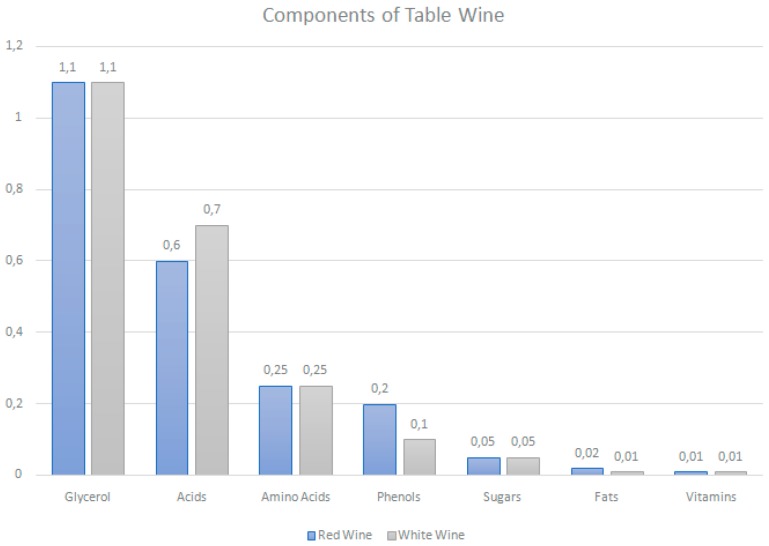
Components of table wine. Estimates of typical gross composition (percentage weight). Phenols constitute the major compositional difference between red and white table wines. Water and ethanol content is similar for both types of wine (87% and 10%, respectively) [26].

Wine is a water-dominant solution containing aldehydes, esters, ketones, lipids, minerals, organic acids, soluble proteins, sugars, vitamins and polyphenols. The latter have been given the greatest attention for their anti-oxidant properties and their ability to act as a free radical terminator and metal chelator (Figure 3) [25].

Wine is categorized into red, white or rosé, based on color, grape variety, sweetness, alcohol content, carbon dioxide content, fermentation, and maturation process or geographic origin [27]. Red wines are obtained by the alcoholic fermentation of musts in the presence of their skins and seeds, whereas white wines are usually produced by the fermentation of grape juice [28]. As most of the polyphenols of grapes are in their skins, red wine is known to contain 10-fold more phenolic compounds than white wine [29]. Indeed, a typical commercial bottle of red wine contains approximately 1.8 g/L of total polyphenols, while the corresponding value for white wine is 0.2 to 0.3 g/L (Table 1) [30,31,32].

Polyphenols play an important role in wine’s bitterness/astringency (proanthocyanidins: polymers of catechins and epicatechins) and color (anthocyanins). The phenolic composition of wine is affected by grape variety, factors that influence the berry development (e.g., soil, geographical location, and weather conditions), winemaking techniques, time of maceration and fermentation in contact with the grape husks and seeds, pressing, maturation, fining, and bottle aging [34]. As the wine ages, and depending on the type of recipient (barrels or bottles) and the stocking temperature, the composition and amounts of different anthocyanins and proanthocyanidins varies, thus changing the color and the astringency/bitterness of wine [35].

Grapes contain non-flavonoid compounds mainly in the pulp, while flavonoid compounds are located in the skins, seeds, and stems [34]. Flavonoids constitute the majority of the phenols in red wine (over 85%) [33]; the main classes of red wine polyphenols include flavonols (quercetin and myricetin), flavanols (catechin and epicatechin), and anthocyanin and stilbenes (resveratrol) [36]. Resveratrol is the most studied stilbene, primarily found in the fresh skin of red grapes (Table 2). Several studies describe the health benefits of this molecule based on its anti-cancer, anti-aging, anti-inflammatory, and anti-oxidant properties [17,22,37,38].

## 4. Red Wine Polyphenols and Cardiometabolic Diseases

In vivo and in vitro studies, animal models, epidemiological data and multiple clinical trials suggest an association between low-to-moderate, regular, alcoholic beverage consumption, particularly red wine, and a lower risk for CVD [8,12,40,41,42]. Furthermore, in a review of ecological, case-control, cohort and clinical studies, authors considered that a moderate consumption of alcoholic drinks can reduce both coronary and all-cause death, with a broad variation in the ranges of moderate intake, with no clear information in ecologic studies, <6 drinks/day to 3–5 drinks/day in case-control studies, <5 drinks/day in cohort studies. One drink represents ~ 14 g of alcohol [43]. However, the relationship between total mortality and consumption of alcoholic beverages follows a U curve [43,44,45], due to the beneficial effects of moderate alcohol intake being even greater than complete abstinence from alcohol, although these effects are lost when consumption is excessive [12]. The benefit on total mortality would occur at doses of approximately 3–30 g/day of alcohol in women and 12–60 g/day in men. Rimm et al. [44] found that drinking 30–50 g of alcohol per day could decrease the risk of ischemic heart disease in men by 42%. Similarly, Stampfer et al. [45] reported that 5–24 g of alcohol intake per day reduced the risk of ischemic heart disease in middle-aged women by 40%, and total stroke by 40–50%. As already mentioned, the dose of moderate alcohol consumption is not specified in all epidemiological studies presented.

Around 50 years ago, epidemiological studies suggested the inverse relationship between alcohol and a lower incidence of CVD [46]. Furthermore, in 1979, St Leger narrowed this association to the consumption of red wine, although alcohol amount was not specified [40]. Later, in 1992, Renaud et al. [8] introduced the concept of the ‘French Paradox’. They described that in France, the intake of saturated fats and serum cholesterol concentration was as high as in the USA or UK, but CVD mortality was much lower, possibly due to the moderate red wine consumption [8]. Since then, several studies showed that red wine—3 to 5 daily glasses—is the most beneficial in reducing the risk of CVD and overall mortality compared with other alcoholic beverages, such as spirits, beer and white wine [47]. The Copenhagen Heart Study showed the inverse association of wine [but not beer or spirits) consumption with CVD, cerebrovascular disease and overall mortality [36]. In another report, Grønbaek et al. [48] followed 24,523 people for a period of 11 years and concluded that low (1–7 drinks/week) and moderate wine drinkers (8–21 drinks/week) had 20% and 24% lower all-cause mortality than non-wine drinkers, respectively.

A large meta-analysis by Rimm et al. [48] in 1996 found that, in most epidemiological studies, wine was superior to other alcoholic beverages, but not in the case-control studies, nor in the prospective studies. Therefore, it was not possible to conclude that one type of alcoholic beverage was superior to another, and the great variability of the approach of the different studies, the amount consumed and the type of drink limited the conclusions of this meta-analysis [48]. However, the authors agreed that the consumption of small quantities of alcohol clearly decreased CVD risk [49].

A review by Mostofsky et al. [50] concluded that regular alcohol intake has both risks and benefits. Women with low-to-moderate intake (defined as up to 1 drink a day) and regular frequency (>3 days/week) had the lowest risk of mortality compared with abstainers and women who consumed substantially more than 1 drink per day [50]. Contradictorily, a systematic analysis published in 2018 reported that the level of alcohol consumption that minimized harm across health outcomes was zero (95% CI 0.0–0.8) standard drinks per week [51], although this was based on studies with inherent limitations. Data from a prospective follow-up (12 years) study of 18,394 Spanish participants, showed that the benefits of wine intake were associated with low-to-moderate consumption (10–50 g/d [men] or 5–25 g/d [women]) on a regular basis, within a Mediterranean pattern, referring mainly to red wine intake with principal foods, and not to binge drinking or regular spirits’ consumption [12].

Overall, although alcohol intake has shown both risks and benefits, the phenolic compounds from red wine exert a favorable effect on improving CVD mortality in different populations, when consumed in low-to-moderate quantities on a regular basis, within a Mediterranean pattern.

### 4.1. Red Wine Polyphenols and Blood Lipids

Moderate alcohol consumption (30 g alcohol/d) can raise high-density lipoprotein cholesterol (HDL-C) concentrations and, until recently, this was thought to be the main cardiovascular protector effect of moderate alcohol intake [52]. Indeed, moderate alcohol consumption (up to one drink or 15 g alcohol a day for women and up to two drinks or 30 g alcohol a day for men, whatever the alcoholic beverage consumed) elevates HDL-C in a dose-dependent manner [53]. In addition, polyphenols affect apolipoproteins (Apo) A and B [54], modify Very Low Density Lipoproteins (VLDL) particles, and reduce plasma triglyceride (TG) levels by increasing the lipoprotein lipase (LPL) activity, which decreases low-density lipoprotein cholesterol (LDL-C) concentrations in the circulation [55]. Ethanol decreases plasma Apo B, whereas red wine (but not gin) increases ApoA-I and II in healthy volunteers [56,57]. Rimm et al. [58] also found a strong and consistent evidence linking moderate alcohol intake with higher concentrations of HDL-C and Apo A-I, as well as a weak association between moderate alcohol consumption and increased TG.

In individuals with dyslipidemia, LDL/HDL ratio was decreased (*p* = 0.05) after red wine consumption for 30 days (125 mL per day in women and 250 mL per day in men) [59]. Similarly, in hypercholesterolemic postmenopausal women, 400 mL/day of red wine consumption for 6 weeks significantly reduced LDL-C by 8% and increased HDL-C by 17% [60]. In patients with well-controlled T2DM, the intake of 150 mL/day of red wine at dinner for two years significantly increased HDL-C and Apo AI levels, and reduced total cholesterol (TC)/HDL ratio [61]. A randomized crossover trial showed that Apo AI, Apo A2 and HDL levels increased in men at high cardiovascular risk who consumed 30 g alcohol/day in the form of red wine for 4 weeks -compared with gin-, supporting a beneficial effect of the non-alcoholic fraction of red wine [52]. Furthermore, a daily glass of red wine (0.1 L women, or 0.2 L men) significantly improved the LDL/HDL ratio in 108 patients with carotid atherosclerosis, even in those on statin therapy [62].

Several studies showed that resveratrol can reduce serum levels of TC, LDL-C, and TG, as well as raise HDL-C [63,64,65,66,67,68,69,70,71]. Nevertheless, other studies reported no effect of resveratrol on serum lipids [26,72,73,74]. Therefore, resveratrol may play a role in CVD prevention, but robust evidence is lacking [63].

Overall, phenolic compounds, present in red wine, can modify the quantity, composition and function of different lipoproteins, thus subsequently affecting CVD risk.

### 4.2. Red Wine Polyphenols and Blood Pressure

Although it is well documented that heavy alcohol consumption is associated with arterial hypertension, low-to-moderate alcohol intake (15–30 g of alcohol) seems to exert a beneficial effect on both BP and CVD [75]. In this context, reduction of alcohol consumption in heavy drinkers led to a dose-response decrease in BP [76].

Red wines and grapes stimulate endothelium-dependent relaxation of vessels via enhanced generation and/or increased biological activity of nitric oxide (NO), leading to elevated cGMP levels [77,78,79,80]. In vivo red wine polyphenols reduced BP in normotensive and hypertensive rats [81,82,83,84]. In T2DM patients, daily consumption of 0.15 L of red wine taken with dinner for 2 years, transiently decreased BP in healthy volunteers at midnight and early in the morning compared with water, without differences in the mean 24 h BP [61]. In several prospective studies, the relationship between red wine consumption and BP is U- or J-shaped, suggesting a slight decrease in BP among those who consume one drink a day [85]. In healthy volunteers, the prolonged effect of wine was different from a control alcohol drink (13.5% alcohol) as wine decreased BP and reduced the complexity of the heart-interbeat interval and ventricular repolarization interval [86]. A randomized trial evaluated the effects of alcoholic and non-alcoholic red wine and gin consumption in 67 men with high cardiovascular risk and showed that dealcoholized red wine decreased systolic and diastolic BP, and these changes correlated with increases in plasma NO [87].

Overall, phenolic compounds from red wine can improve both systolic and diastolic BP in different populations, when consumed at low doses.

### 4.3. Molecular Mechanisms of the Effects of Red Wine Polyphenols on the Atheromatous Plaque

The majority of CVD events originate from atherosclerosis [88]. Atherosclerosis is a low-grade inflammatory and oxidative disease; cell and endothelial expression of adhesion molecules and chemokines participate in the recruitment of circulating leukocytes to the vascular endothelium and their migration into subendothelial spaces [87].

Several experimental studies identified endothelial dysfunction as the initial event in hypercholesterolemia, resulting in increased endothelial permeability to lipoproteins and other plasma components [89]. Red wine polyphenols can promote endothelial-dependent vasodilation by acting on NO enhancement and release [90,91].

It is now well known that inflammation plays a role in atherogenesis [92,93]. Resveratrol inhibits the activity of inflammatory enzymes (cyclooxygenase and lipoxygenase) [94,95] and inhibits the production of interleukin (IL)-1 [96], IL-2, IL-12, interferon-γ (IFN-γ), and tumor necrosis factor-α (TNF-α) [97,98,99], IL-6 [100], IL-4 [101] and IL-8 [102]. Resveratrol also attenuates proinflammatory transcription factors and activator protein-1 (AP-1) [103].

A moderate intake of red wine can prevent nuclear factor-kappa B (NF-kB) activation in peripheral mononuclear cells in healthy volunteers even after a saturated fat enriched breakfast [104]. Red wine polyphenols have also been demonstrated to counteract monocyte and leukocytes adhesion to the endothelium by downregulating the expression of proatherosclerotic and prothrombotic factors, such as vascular cell adhesion molecule 1 (VCAM1), intercellular adhesion molecule 1 (ICAM1), [105] as well as monocyte chemoattractant protein-1 (MCP-1) [106]. Red wine polyphenols (catechin and quercetin) inhibit LDL oxidation, thus attenuating the development of atherosclerosis [107,108,109,110].

Resveratrol also reduces reactive oxygen species (ROS) generation in cardiac tissues of guinea pig [111,112], as well as hydrogen peroxide production by ox-LDL in murine macrophages [113]. It exerts a potent antiproliferative activity on vascular smooth muscle cell (VSMC) [114,115] and inhibits PDGF-receptor mitogenic signaling in mesangial cells [116]. In this context, trans-resveratrol can inhibit PDGF-stimulated DNA synthesis and cell proliferation in cultured VSMC [117].

Platelet aggregation has been implicated in atherogenesis. The mechanisms involved include generation of ROS by activated platelets [118,119]. Interestingly, resveratrol inhibits human platelet aggregation both in vitro and in vivo [72].

Overall, red wine polyphenols can promote endothelial-dependent vasodilation, inhibit the activity of inflammatory enzymes and the production of several types of proinflammatory and oxidant mediators, thus attenuating the development of atherosclerosis.

### 4.4. Red Wine Polyphenols and Glucose Metabolism

Several observational and prospective randomized trials reported a strong association between hyperglycemia and poor clinical outcomes with regard to mortality, morbidity, length of hospital stay, infections, and overall complications [120]. Identification of modifiable lifestyle interventions, including dietary factors, that can reduce the incidence of T2DM, is an important area of research [121,122].

One dietary factor of interest is polyphenol-rich food consumption, since dietary polyphenols have been shown to lower the risk of T2DM [122,123]. Red wine polyphenols can beneficially affect insulin resistance and lipoprotein plasma concentrations [52].

In diabetic animals, resveratrol improved glucose homeostasis, reduced blood glucose levels, protected pancreatic β-cells and increased insulin secretion [124,125]. Similarly, resveratrol decreased insulin resistance in T2DM patients [126,127], obese men [128], older adults with insulin resistance [129], and patients with metabolic syndrome (MetS) [130]. The effective doses of resveratrol fall into a wide range, from 10 to 1 g per day per person [125,126]. In addition, resveratrol improved β-cell function in T2DM patients [127], and decreased BP in T2DM patients [127,131] and in obese men with insulin resistance [128], and reduced diabetic ulcer size [132]. Moreover, a single 75 mg dose of resveratrol was shown to improve neurovascular coupling and cognitive performance in T2DM patients [133]. However, in a recent double-blind, randomized, placebo-controlled trial, supplementation with 40 or 500 mg/day resveratrol did not improve the metabolic pattern of T2DM patients [134]. In addition, a double-blind, randomized and crossover study, found that 5 weeks of resveratrol treatment (500 mg twice daily) had no effect on the secretion of glucagon-like peptide 1 (GLP-1), gastric emptying or glycemic control in 14 diet-controlled T2DM patients [135].

Overall, there is little evidence regarding the effect of wine polyphenols on glucose and insulin metabolism. Further trials are required to better understand the antidiabetic properties of resveratrol, as well as to establish the therapeutic potential of other stilbenoids in T2DM patients.

## 5. Polyphenols Content in Olive Oil

Olive oil and its different variants, virgin and extra virgin, are a symbol of the Mediterranean Diet. In all the traditional forms of this diet found in the Mediterranean area, virgin olive oil is obtained directly from olives, the fruit from *Olea europeae* tree, and by mechanical extraction, being a natural juice that constitutes the main source of fat from this dietary pattern. Olive oil is composed by glycerol fraction (90–99%) and non-glycerol (0.4–5%) [136]. One of the main characteristics of olive oil is the high content of monounsaturated fat and a low concentration of saturated fat. The main monounsaturated fat, oleic acid, represents 70–80% of the fatty acids present in olive oil and is the responsible of many health-promoting properties with effects that include a reduction in CVD, neurodegenerative diseases and cancer. Moreover, despite the content of monounsaturated fat, virgin olive oil contains a wide variety of bioactive compounds, that change between olives and the different virgin olive oils available for consumption. Polyphenols are probably the most relevant of these bioactive compounds [137]. Olive oil phenols include simple phenolic compounds (vanillic, gallic, coumaric, caffeic acids, tyrosol and hydroxytyrosol) and complex compounds like the secoroids (oleuropein and ligstroside), and the lignans (1-acetoxypinoresinol and pinoresinol) [136]. The concentration of polyphenols in olive oil is between 40 and 1000 ppm and is the result of an interaction of various factors including the olive cultivar, time to maturation, the climate or the extraction process [138].

## 6. Olive Oil Polyphenols and Cardiometabolic Diseases

The concept of the MedDiet is heterogeneous, comprising slightly different dietary patterns, mainly depending on the local preferences of the Mediterranean location. This composition includes a low saturated fat and a high monounsaturated fat content (mainly from oleic acid in olive oil).

Only extra virgin and virgin olive oils contain a significant percentage of phenols, since they are derived from the physical pressure of the olives when obtaining the oil. Olive oil has at least 30 phenolic compounds. Along with the phenols, as previously mentioned, several non-fatty minor components of great biological potential, including vitamin E, carotene or chlorophyll, make virgin olive oil a unique, nutraceutical product. Some of the healthy benefits of olive oil on cardiometabolic diseases may be due to these other compounds, although there is no evidence to ascribe a certain effect to one specific compound.

There is evidence linking phenolic compounds present in virgin olive oil with traditional and non-traditional CVD risk factors [139].

### 6.1. Olive Oil Polyphenols and BP

Arterial hypertension is a major risk factor for CVD, especially in elderly people [51]. The MedDiet is associated with a decrease in both systolic and diastolic BP [140]. The effect of virgin olive oil phenols on BP was described by Fito et al. [141], who reported a reduction in systolic BP after high-phenolic olive oil consumption, that was not reproduced after low-phenolic refined olive oil, in hypertensive patients with stable CHD. Moreno-Luna et al. [142] compared the effect of interventions with polyphenol rich virgin olive oil and high-oleic sunflower oil (polyphenol-free) in hypertensive women; the virgin olive oil rich diet lowered systolic and diastolic BP. In a substudy of the PREDIMED trial, in which the relationships between polyphenol intake, circulating inflammatory biomarkers and CVD risk factors were evaluated in elderly individuals, the investigators reported that high polyphenol intake improved CVD risk factors, and BP and lipid profile [143].

A recent study evaluated the effects of oral supplementation with hydroxityrosol on early atherosclerosis markers in middle-aged healthy adults [143]. Hydroxityrosol showed anti-atherosclerotic properties by improving endothelial function, BP and circulating oxidized LDL levels [144].

Overall, phenolic compounds from virgin olive oil have shown a favorable effect on both systolic and diastolic BP in different populations.

### 6.2. Olive Oil Polyphenols and Lipids

Plasma TC, LDL-C and HDL-C levels are related to CVD risk, and, hence, they are included in CVD risk assessment tools [145]. Substituting sunflower oil with olive oil can reduce LDL-C and increase HDL-C concentrations [146]. TGs are recognized as a potent CVD risk factor. Furthermore, postprandial lipemia, that involves TG and TG rich lipoproteins, is also a major CVD risk factor [147,148,149,150,151,152,153,154,155,156,157].

Polyphenols in virgin olive oil may modulate lipids and their metabolism. A double-blind, crossover, controlled trial, with hypercholesterolemic patients evaluated the particle size for VLDL, LDL and HDL groups by nuclear magnetic resonance and their corresponding serum levels after the intake of natural virgin olive oil and two different functional virgin olive oils, with a concentration of phenolic compounds of 500 and 250 ppm, respectively [158]. Olive oil phenols beneficially affected lipoprotein particle atherogenic ratios and subclasses’ profile distribution, concluding that polyphenol-enriched olive oil can enhance the olive oil’s healthy properties while consuming the same amount of fat.

Long-term consumption of virgin olive oil with different concentrations of phenols may slightly increase HDL-C levels [159]. Although elevated HDL-C is a protective factor for CVD, drug trials raising HDL-C failed to lower CVD risk [160]. This interesting finding may imply that only natural ways of improving HDL-C are effective. Furthermore, it supports the hypothesis [161] that HDL composition and function might be more important to CVD outcomes than the amount of HDL-cholesterol [162,163]. In this context, Pedret et al. [164] showed that the consumption of virgin olive oil or phenol-enriched virgin olive oils affected the HDL proteome in a cardioprotective model by up-regulation of proteins related to cholesterol homeostasis oxidation and hemostasis and by down-regulation of proteins involved in lipid transport and immune response. Fatty acid and phenol compounds composition were major contributors to HDL remodeling.

The EUROLIVE study, that included 200 patients from six different European countries, was the first international study with a large sample size that evaluated the effects of the phenolic compounds present in olive oil on plasma lipid levels. Investigators designed a crossover study [165], where participants were randomly assigned to three sequences of daily administration of 25 mL of three olive oils with low, medium or high phenolic content. A linear increase in HDL-C levels and a decrease in TC-HDL ratio was observed for low-, medium-, and high-phenol olive oil. In addition, TG levels decreased with all olive oils and LDL-C was reduced with the medium and high phenol ones. Another interesting finding of the EUROLIVE study was related to oxidized LDL (oxLDL), an immunogenic particle that plays a key role in the development of atherosclerosis; a protective role of OxLDL autoantibodies (OLAB) has been proposed. Their findings showed that OLAB concentrations, adjusted for oxLDL, increased in a dose-dependent way with the polyphenol content of the olive oil administered [166]. Another study demonstrated that the consumption of olive oil phenols decreased LDL-C levels and LDL atherogenicity in healthy young men [167].

Overall, polyphenolic compounds present in virgin olive oil can modify not only the quantity, but also the quality of different lipoproteins.

### 6.3. Phenolic Compounds, Obesity, MetS and T2DM

During the past few decades, the burden of obesity has become a major public health challenge and strategies to establish dietary patterns to reduce obesity, MetS and T2DM are now a priority.

Evidence shows that the MedDiet can prevent metabolic diseases such as obesity, MetS and T2DM [168,169,170,171]. In this context, a meta-analysis including 16 randomized clinical trials found that the MedDiet led to weight loss, especially when patients adhere to other healthy lifestyle habits [171]. In addition, an inverse correlation between the MetS and adherence to MedDiet has been reported [168,169]. Since T2DM is frequent in patients with MetS, it is reasonable to infer that MedDiet might prevent T2DM development or improve the impaired metabolic status [170]. Based on current evidence, the American Diabetes Association [172] and an international panel of lifestyle recommendations for prevention and management of the MetS [173,174] suggest that this dietary pattern is useful to prevent these metabolic diseases.

A recent meta-analysis reported that increased adherence to MedDiet significantly reduced CVD, stroke and CHD morbidity and mortality in T2DM patients [175].

In the PREDIMED trial, the effects of a MedDiet supplemented with extra virgin olive oil on glucose metabolism were evaluated [7,176,177]. In particular, a MedDiet enriched with extra virgin olive oil lowered the risk of T2DM by 40% (HR: 0.60; 95% CI: 0.43, 0.85) in patients with a high CVD risk compared with the control group [177]. Other reports also from the PREDIMED trial, showed a reduction of new-onset T2DM in elderly individuals with the highest intake of phenols [178] and an inverse correlation between obesity and high phenol consumption [179]. Different mechanisms and pathways may be responsible for these beneficial effects. Virgin olive oil polyphenols may influence glucose metabolism through the inhibition of carbohydrate digestion and absorption, reduction of glucose release from the liver, stimulation of glucose pathways in peripheral tissues, production of advanced glycosylated end products [123,180,181] and prevention of abnormal postprandial lipemia. In this context, the elevated postprandial lipemia present in T2DM patients and in patients with MetS can be influenced by diet. A report from the CORDIOPREV trial, that included 557 high cardiovascular risk patients, evaluated the influence of two dietary patterns (a MedDiet rich in extra virgin olive oil vs a low-fat diet) on postprandial lipemia, showing that the long-term consumption of a MedDiet rich in virgin olive oil improved postprandial lipemia mainly in T2DM patients [182].

The effect of extra virgin olive oil consumption on the need for glucose lowering medications has also been evaluated. In the PREDIMED study, participants that followed a MedDiet rich in extra virgin olive oil delayed the initiation of antidiabetic drug therapy compared with the other dietary patterns [183].

Overall, evidence supports that a MedDiet rich in extra virgin olive oil, an important source of phenols, and may prevent obesity, MetS and T2DM via multiple metabolic pathways, reinforcing the key role of diet in the treatment and prevention of these metabolic disorders.

### 6.4. Olive Oil Polyphenols and Endothelial Function

The endothelium has been traditionally considered a monolayer that covers the wall of blood vessels, acting as a barrier that separates vascular light from the rest of the structure of the blood vessel and tissues. Over the years, this concept has undergone a great evolution, with endothelium now being considered a dynamic and complex endocrine, autocrine and paracrine organ with multiple functions, responsible for maintaining vascular homeostasis through various interactions between endothelial cells and vascular lumen [184].

Endothelial dysfunction is defined as an alteration of the physiology of the endothelium that predisposes to inflammation, vasoconstriction and increased vascular permeability, facilitating platelet aggregation, thrombosis and arteriosclerosis, representing a key early step in the development of atherosclerosis, participating in the progression of plaque and the appearance of atherosclerotic complications [185,186,187,188].

The high content of phenolic compounds present in extra virgin olive oil may slow the atherogenic process by inhibiting oxidative damage and restoring endothelial function. In vitro and cell cultures studies showed that phenolic compounds possess antioxidant, anti-inflammatory and antithrombotic properties [189,190]. Later, in vivo studies found that the concentration of phenolic compounds can modulate endothelial function: in a study with 21 hypercholesterolemic patients, when phenolic acid content was decreased from 400 to 80 ppm, endothelial function was impaired as assessed by ischemic reactive hyperemia (IRH) in fasting and postprandial states [191]. Furthermore, Valls et al. [192] showed that functional virgin olive oil intake improved postprandial endothelial function (determined by IRH) in a linear trend from baseline to 5 h compared with virgin olive oil. Of note, hydroxytyrosol was the main biological metabolite and it increased in a dose-dependent manner with the polyphenol content of the extra virgin olive oil.

As previously mentioned, a recent study showed that oral supplementation with hydroxityrosol in middle-aged healthy adults exerted anti-atherosclerotic effects by improving endothelial function [144]. In particular, hydroxityrosol may exert its vasculoprotective effects via the activation of a nuclear factor-E (2)-related factor-2 (Nrf2) pathway that increases the expression of other antioxidants, like NAD(P)H: quinone oxidoreductase 1 [193,194].

Different polyphenols from the MedDiet may have synergic effects. Karatzi et al. [195] reported that high-phenol extra virgin olive oil combined with red wine induced a favorable effect on postprandial flow-mediated dilatation in healthy young men. In a recent report from the CORDIOPREV study, MedDiet improved flow mediated vasodilation after 1.5 years [196].

Another important point refers to genes that can interact with extra virgin olive oil diet phenols to regulate endothelial function. In this context, individuals with variations in the NO synthase gene (Glu298Asp polymorphism) had a worse postprandial endothelial function [197]; this was improved with a meal based on extra virgin olive oil with a high content of phenols [198].

Overall, evidence shows that virgin olive oil phenols may improve endothelial function in large, medium and small size vessels, indicating the involvement of various mechanisms, including a higher NO bioavailability, upregulation of antioxidant pathways and a decrease in pro-oxidant substances production. Finally, gene–diet interactions have also been suggested.

### 6.5. Olive Oil Polyphenols: Inflammation, Oxidative Stress and Hemostasis

The pathogenesis of atherosclerosis is characterized by a low-grade chronic inflammation, caused by monocytes and other important inflammatory markers, including cytokines, neutrophils or natural killer cells [199]. Several studies have reported a direct association between the consumption of MedDiet and improved inflammation, oxidative stress and hemostasis [4,200,201,202]. Certain diet micronutrients are responsible for these effects [203,204,205]. In this context, extra virgin olive oil phenol fraction improves the hemostatic profile during postprandial state [206] and enhances endothelial function by reducing the redox state [191]. The expression of inflammatory genes is lower when the virgin olive oil used in meals is rich in polyphenols, compared with other diets [204]. In this context, the consumption of virgin olive oil rich in phenolic compounds was shown to reduce the risk of atherosclerosis by decreasing inflammation and improving the antioxidant profile in the vascular endothelium, inducing a decrease in MCP-1 and CAT gene expression [207].

Experimental studies reported a favorable effect of polyphenols in the virgin olive oil, and in other oils to which these polyphenols had been added. In a study with 20 obese people, who received breakfast based on oils after several heating cycles, postprandial oxidative stress and DNA oxidation damage were decreased after the breakfast enriched with virgin olive oil or seed oil with added polyphenols, compared with the breakfast prepared with sunflower or seed oil with an added artificial antioxidant (dimethylpolysiloxane) [201]. These results suggest that frying oils rich in phenolic compounds are a healthier alternative for frying [208]. Although the specific influence of polyphenols on hemostasis has not been properly studied, MedDiet, or meals enriched with virgin olive oil, have been reported to lower the procoagulant state [209,210]. This fact has been related to platelet function [211,212,213,214,215,216,217,218,219] or fibrinolysis [218].

Overall, polyphenols in olive oil can improve inflammation, oxidative stress and hemostasis, thus potentially preventing atherogenesis.

### 6.6. Phenolic Compounds and Gut Microbiota

In the last decade, microbiota has emerged as a novel pathway linked to CVD and non-cardiovascular diseases [220,221,222,223]. There is enough evidence to consider gut microbiota as a new marker of CVD, related to the major traditional CVD risk factors such as T2DM, arterial hypertension, dyslipidemia and obesity [222]. The gut microbiome effects can be altered by diet in a variable and complex way. Research in this field is focused on understanding the different pathways involved in the gut microbiota response to identify subjects at risk of developing CVD or metabolic disorders, as well as to apply personalized dietary interventions. Some of the reported gut microbiota-related mechanisms by which gut bacteria could influence CVD risk include the combination of short fatty acids generation, reduction in cholesterol available for reabsorption and production of active metabolites with cardioprotective properties [224].

Currently, no large studies on the effect of phenol compounds in olive oil on microbiota have been conducted. In the CORDIOPREV study, gut microbiota at baseline and after 2 years of dietary intervention were analyzed in 106 CHD patients, showing that the MedDiet rich in virgin olive oil partially restored the gut microbiome dysbiosis in obese patients [173]. Furthermore, another report from the same study showed that MedDiet can modulate gut microbiota, leading to an improvement in insulin sensitivity [225].

## 7. Polyphenols in Olive Oil and Red Wine and Nonalcoholic Fatty Liver Disease

Non-alcoholic fatty liver disease (NAFLD) is considered to be the hepatic component of the metabolic syndrome [226,227], due to its strong association with insulin resistance and obesity. Inflammation and oxidative stress are the major risk factors involved in its pathogenesis [228]. It has become an emerging public health problem worldwide due to its increasing prevalence [229], and it presents a wide range of liver damage that may lead to severe liver disease, such as cirrhosis and hepatocellular carcinoma [230]. Many of the genetic factors predisposing to NAFLD suggest a critical role for the lipid metabolism and inflammation, which ultimately affect intracellular oxidative processes [231,232].

Lifestyle interventions based on exercise and a balanced diet are considered the cornerstone of NAFLD management [7]. The EASL-EASD-EASO clinical Practice Guidelines have recently encouraged MedDiet as a lifestyle choice for treating this condition [233]. The beneficial effects of the MedDiet on the progression of the NAFLD are believed to be due do the polyphenol’s antioxidant and anti-inflammatory properties.

Regarding the specific effects of wine and olive oil phenols on NAFLD, there are very few data available. A study conducted in rats by Lama et al. showed that polyphenol-rich virgin olive oil limits high fat diet-induced insulin resistance, inflammation, and hepatic oxidative stress, preventing nonalcoholic fatty liver disease progression [234]. Nuclear factor-erythroid 2-related factor 2 (Nrf2) is the main transcription factor which maintains cellular redox status through downstream modulation of antioxidant defense genes [235]. Rubio-Ruiz et al. [236] demonstrated that a mixture of resveratrol and quercetin improved the antioxidant capacity and increased the expression of *Nrf2* in a rat model of metabolic syndrome. Supplementation with quercetin and resveratrol have been reported to reduce lipid peroxidation in both the liver [237] and serum [238] of NAFLD animals. Gomez-Zorita et al. [237] also reported a reduction in fatty acid availability and Bujanda et al. [239] reported an increase in the catalase, superoxide dismutase and glutathione peroxidase enzymatic activities in the liver of NAFLD animals fed with resveratrol. Other studies using quercetin and resveratrol suggested that their anti-inflammatory effect was achieved through the repression of NF-κB translocation or gene expression as well as a reduction in the JNK phosphorylation protein levels [238,240]. Also, enhanced adiponectin secretion and gene expression induced by polyphenol-rich grape extract [241] may also contribute to the reduction in hepatic inflammation and ultimately the progression of NAFLD.

The effects of polyphenols have been explored in some clinical trials, with polyphenols not derived from olive oil, wine or grapes. Chang et al. [242] evaluated the effects of 150 mg/day of polyphenols composed of 1.43% flavonoids, 2.5% anthocyanins and 1.7% phenolic acids compared to placebo in overweight NAFLD patients. After 12 weeks of treatment, a significant 15% reduction in fatty liver score was observed in the polyphenol group, with no changes in AST or ALT levels, but decreases in body weight, body mass index, body fat and waist-to-hip ratio were also observed. Guo et al. [242] explored the benefits of 250 mL of bayberry juice, meaning 1350 mg/day of polyphenols—phenolic acids and anthocyanins or placebo twice daily—for four weeks in young NAFLD patients. No significant differences in serum levels of ALT and AST were appreciated between the groups, but there was a reduction in serum levels of hepatocytes apoptosis biomarkers, namely CK-18 and tissue polypeptide-specific antigen. In two other clinical trials the effects of specific polyphenols, anthocyanins and catechins were evaluated. Suda et al. [243] assigned overweight NAFLD men to consume two bottles of purple sweet potato beverage, where the phenolic compounds represented by acylated anthocyanins were 400 mg/day, or placebo. After 8 weeks, the intake of phenolic compounds significantly reduced the serum levels of ALT versus placebo. Sakata et al. [244] investigated the effects of green tea with high-density catechins in overweight NAFLD patients, randomized to consume different amounts of catechins (0, 200, or 1080 mg/day) for 12 weeks in a cup of 700 mL/day. The consumption of the highest dose of catechins significantly decreased serum ALT levels by 42% and improved liver fat content with a liver-to-spleen CT attenuation ratio that increased from 92% to 102%. Another five clinical trials have been conducted with resveratrol, but results were controversial [245].

In summary some promising data from animal and in vitro studies, as well from some short clinical trials, suggest that polyphenols could play a role in the management of NAFLD, thus collaborating to the beneficial effect of MedDiet on NAFLD.

## 8. Conclusions

**Figure 4:**
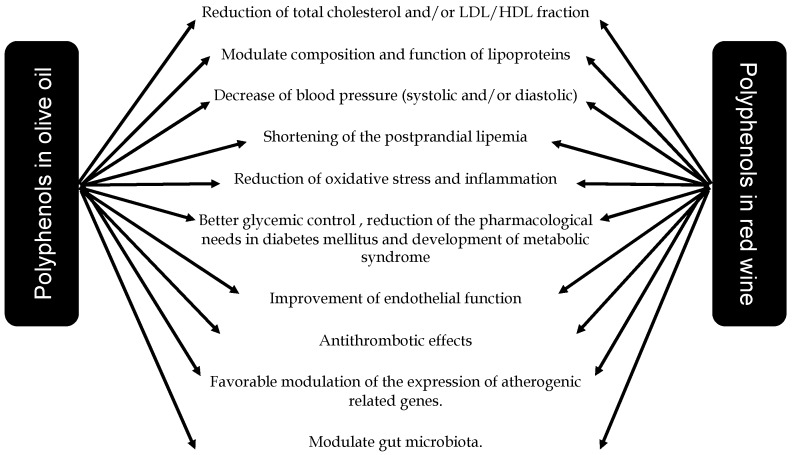
Impact of polyphenol content in moderate consumption of wine and olive oil on cardiovascular disease prevention and management.

Low-to-moderate consumption of red wine with meals and virgin olive oil have been reported to prevent cardiometabolic diseases, including CVD, T2DM, MetS and obesity. Current mechanisms underlying the beneficial effects of the MedDiet include a reduction in inflammatory and oxidative stress markers, improvements in lipid profile, insulin sensitivity and endothelial function, as well as antiatherosclerotic and antithrombotic properties, mainly attributable to their polyphenols content (Figure 4).

In 2004 and 2011, the FDA and the European Food Safety Authority, respectively, authorized a health claim for olive oil (and its phenolic compounds) consumption to prevent CHD, based on its effects on the lipid profile [139]. Consuming low-to-moderate amounts of red wine within the principal meals, in a Mediterranean style, can also exert cardioprotection.

## Figures and Tables

**Table 1:** Content of majority phenolic compounds of red and white wines, expressed in milligrams of gallic acid equivalent (mg/GAE/L). Adapted from Markoski MM, 2016 [33].

Phenolic Compounds	Red Wine (mg/GAE/L)	White Wine (mg/GAE/L)
Catequin	191	35
Epigallocatechin	82	21
Gallic Acid	95	7
Cyanidin-3-glucoside	3	0
Malvidin-3-glucoside	24	1
Rutine	9	0
Quercetin	8	0
Myricetin	9	0
Caffeic acid	7.1	2.8
Resveratrol	1.5	0
Total content of phenolics	2567	239

**Table 2:** The phenolic compounds in different parts of grape and its products. Adapted from [39].

Resource	Phenolic Compounds
Seed	gallic acid, (+)-catechin, epicatechin, dimeric procyanidin, proanthocyanidins
Skin	Proanthocyanidins, ellagic acid, myricetin, quercetin, kaempferol, trans-resveratrol
Leaf	myricetin, ellagic acid, kaempferol, quercetin, gallic acid
Stem	rutin, quercetin 3-O-glucuronide, trans-resveratrol, astilbin
Raisin	hydroxycinnamic acid, hydroxymethylfurfural

## Abbreviations

Apo	Apolipoproteins
BP	blood pressure
CVD	Cardiovascular disease
GLP-1	glucagon-like peptide 1
HDL-c	High-density lipoprotein cholesterol
LDL-c	low-density lipoprotein cholesterol
LPL	Lipoprotein lipase
MedDiet	Mediterranean style diet
NO	nitric oxide
ROS	Reactive Oxygen Species
RWPs	Red Wine Polyphenols
TC	Total cholesterol
TG	triglyceride
T2D	Type 2 diabetes
VLDL	Very Low-Density Lipoproteins
